# Spontaneous common bile duct perforation due to choledocolithiasis accompanied with pancreaticobiliary maljunction in an adult: a case report

**DOI:** 10.1186/s40792-021-01290-9

**Published:** 2021-09-08

**Authors:** Risa Sakamoto, Kengo Kai, Masahide Hiyoshi, Naoya Imamura, Koichi Yano, Takeomi Hamada, Takahiro Nishida, Fumiaki Kawano, Daichi Sakurahara, Yukako Uchise, Koji Yamamoto, Hiroaki Kataoka, Atsushi Nanashima

**Affiliations:** 1 Department of Surgery, University of Miyazaki Faculty of Medicine, 5200 Kihara, Kiyotake, Miyazaki, 889-1692 Japan; 2 Department of Pathology, University of Miyazaki Faculty of Medicine, 5200 Kihara, Kiyotake, Miyazaki, 889-1692 Japan

**Keywords:** Spontaneous common bile duct perforation, Pancreaticobiliary maljunction, Congenital biliary dilation, Diverticulum-like change, T-tube drainage

## Abstract

**Background:**

Spontaneous common bile duct (CBD) perforation is an extremely rare disease in adults. We report an adult case of CBD perforation due to choledocolithiasis accompanied with pancreaticobiliary maljunction, which is, to our knowledge, the first such case report based on a search using PubMed.

**Case presentation:**

A 71-year-old woman with consciousness disorder was transported to the emergency department of another hospital. She was diagnosed as having severe peritonitis with septic shock and transferred to our hospital for emergency surgery. Enhanced computed tomography (CT) revealed supraduodenal CBD dilation similar to a diverticulum and a defect of bile duct wall continuity. Furthermore, CT showed a long common channel of the pancreaticobiliary duct, so she was diagnosed as having spontaneous CBD perforation with pancreaticobiliary maljunction. Emergency surgery was performed that revealed a necrotic diverticulum-like change on the supraduodenal part, and a 2.5 × 1 cm perforation was found on the anterolateral wall of the CBD. Peritoneal lavage was performed, and CBD perforation was resolved with a T-tube. The patient suffered refractory intra-abdominal and retroperitoneal abscess formation and bleeding from the abdominal wall, which required a long period of postoperative management. The T-tube was removed on day 136, and the patient was transferred on day 153.

**Conclusion:**

The cause of CBD perforation is commonly considered to be increased intraductal pressure or weakness of the bile duct wall. In this case, pancreaticobiliary maljunction may have significantly influenced onset and the postoperative course. This case suggests that early surgical intervention and appropriate drainage are important to ensure survival.

## Background

Spontaneous common bile duct (CBD) perforation has been described as a perforation of the CBD without traumatic or iatrogenic injury [1]. It is rarely seen in infants and children with choledochal cyst and pancreaticobiliary maljunction and is extremely rare in adults. According to past reports of adult cases, it may be related to either single or multiple factors such as obstruction by a confluent stone [1–10] or tumor infiltration [11], infective necrosis [12], and increased intraductal pressure [11]. We report an adult case of spontaneous CBD perforation due to choledocolithiasis accompanied with pancreaticobiliary maljunction, previous reports of which were not found in a search using PubMed (United States National Library of Medicine, pubmed.ncbi.nlm.nih.gov). Thus, this report includes some important and clinically significant information.

## Case presentation

A 71-year-old woman was transported to the emergency department of another hospital because of consciousness disorder. Enhanced computed tomography (CT) showed an amount of free fluid in the peritoneal cavity mainly around the right upper abdomen without free air. Ultrasonography identified cholecystolithiasis. Paracentesis revealed intra-abdominal bilious fluid with high levels of total bilirubin (21.6 mg/dL) and amylase (8697 U/L) on biochemical examination. She was diagnosed as having severe peritonitis with septic shock and was transferred to our hospital for emergency surgery and intensive care management.

**Fig.1:**
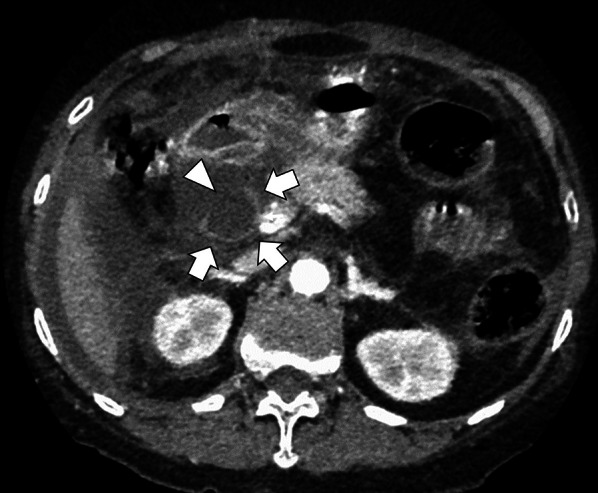
Preoperative computed tomography (axial view). Enhanced computed tomography showed a dilated common bile duct (arrow) with a partial defect in continuity (arrowhead) and peritoneal fluid around the liver

**Fig.2:**
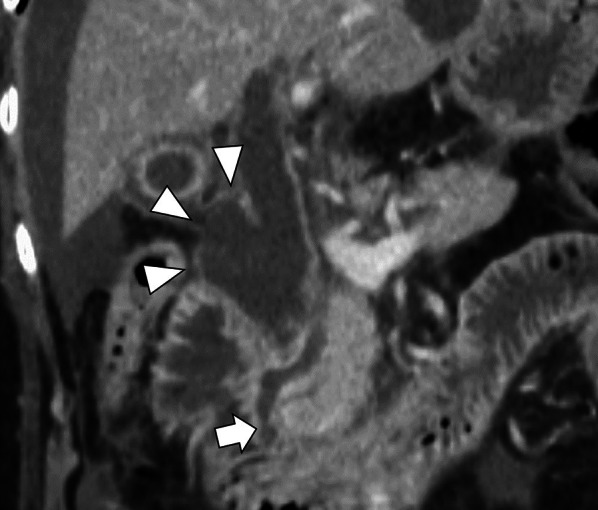
Preoperative computed tomography (coronal view). The common channel of the pancreaticobiliary duct (arrow) was long at 9.3 mm in length and seemed to be joined outside the muscular layer of the duodenal papilla on the enhanced computed tomography coronal view. The dilated common bile duct was accompanied by a diverticulum-like change (arrowhead)

She had no history of past abdominal operations, and other past medical history included choledocholithiasis and pancreatitis. Hematological investigations on admission revealed coagulopathy, renal dysfunction, and circulatory insufficiency (Table 1), which indicated septic disseminated intravascular coagulation. Significantly high levels of serum transaminases, bilirubin, and pancreatic enzymes suggested a condition associated with biliary tract disease. As shown in Fig. 1, enhanced CT revealed that the supraduodenal CBD was markedly dilated similar to a diverticulum (arrow), and the bile duct wall had a partial defect in continuity (arrowhead). Moreover, the common channel of the pancreaticobiliary duct was long at 9.3 mm in length and seemed to be joined outside the muscular layer of the duodenal papilla (arrow) on the coronal CT view (Fig. 2). Eventually, we diagnosed biliary panperitonitis due to the spontaneous CBD perforation accompanied with congenital biliary dilatation and pancreaticobiliary maljunction. As her general condition improved following adequate primary resuscitation, she was able to undergo an emergency laparotomy to cure her septic peritonitis.

**Table 1:** Hematological investigations on admission

Characteristics	Value	Normal value
Complete blood cell count		
White blood cell count, × 10^3^/μL	3.4	3.3–8.6
Hemoglobin, g/dL	14.3	11.6–14.8
Platelet count, × 10^4^/μL	21.5	15.8–34.8
Coagulation/Fibrinolysis Examination		
Prothrombin time, sec	21.1	70–140
D dimer, μg/mL	39	− 1
Fibrinogen/fibrin degradation products, μg/mL	85.6	− 5
Biochemical Examination		
Total bilirubin, mg/dL	3.5	0.4–1.5
Direct bilirubin, mg/dL	2.4	− 0.3
Aspartate aminotransferase, IU/L	1531	8–40
Alanine aminotransferase, IU/L	780	5–40
Amylase, IU/L	970	37–125
Lipase, IU/L	337.6	9–55
Creatinine, mg/dL	2.9	0.4–0.9
Lactate, mmol/L	5.1	0.5–2.0

**Fig.3:**
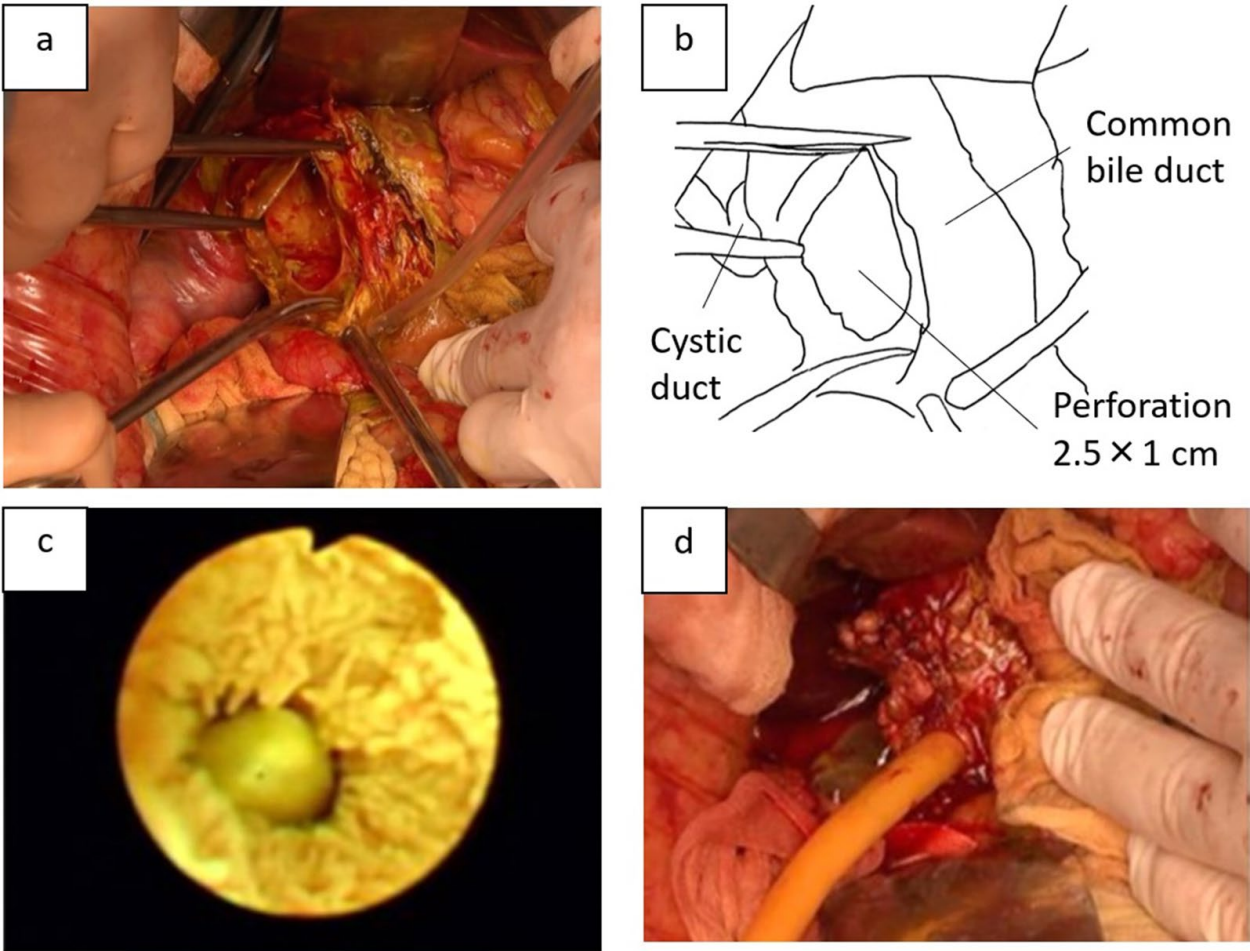
Operative findings. Intraoperative findings revealed that a necrotic diverticulum-like change with bile leakage was present on the supraduodenal part. After removing the necrotic lesion, we found a single 2.5 × 1 cm perforation on the anterolateral wall of the common bile duct (**a**). The above findings are shown in the schema (**b**). Intraoperative cholangioscopy revealed an impacted stone in the major duodenal papilla (**c**). A T-tube was inserted through the perforation (**d**)

**Fig.4:**
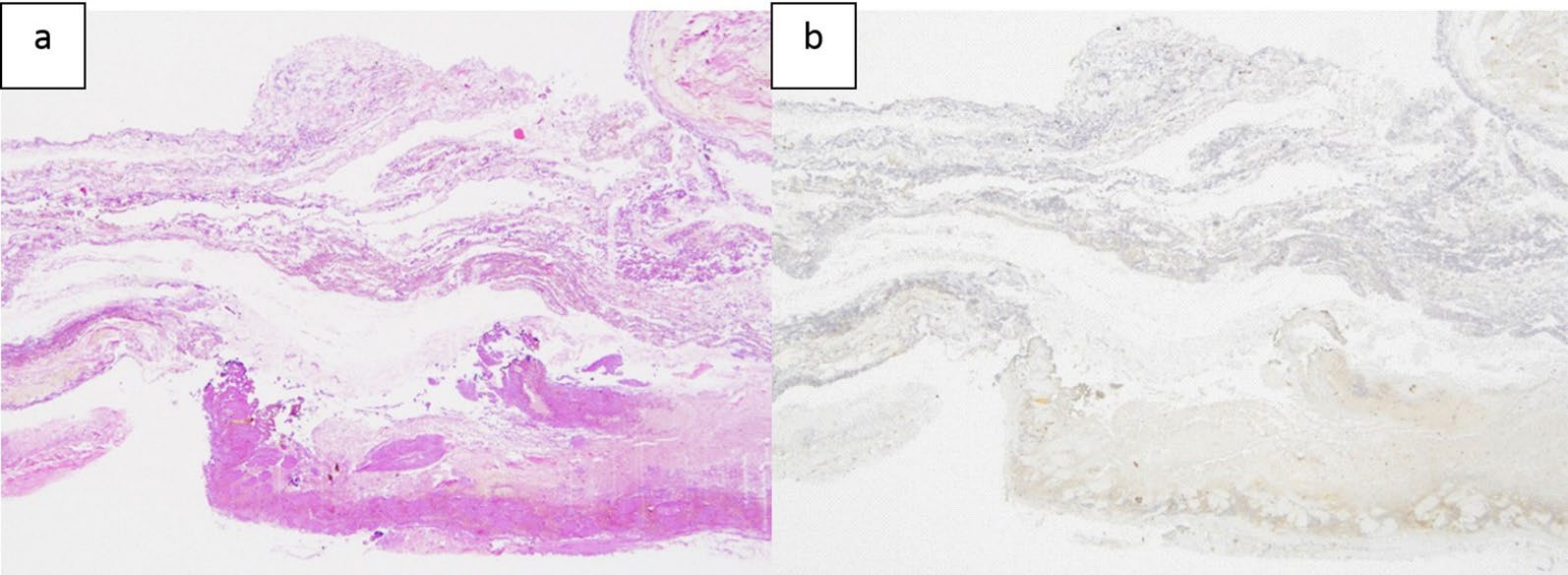
Pathological findings. Hematoxylin and eosin staining revealed that the resected specimen of the common bile duct wall was destroyed that the muscular layer lacked continuity (**a**). Immunohistochemical examination with anti-desmin antibody did not show the presence of smooth muscle in the tissue (**b**)

Intraoperative findings revealed a large amount of bilious ascites along with edematous omentum. No perforation was apparent either in the gallbladder or the gastrointestinal tract. A necrotic diverticulum-like change with bile leakage was present on the supraduodenal part of the CBD. After the necrotic lesion was removed, a 2.5 × 1 cm perforation was found on the anterolateral wall of the CBD, below the junction of the common hepatic duct and cystic duct (Fig. 3a, b). Intraoperative cholangioscopy revealed an impacted stone in the major duodenal papilla (Fig. 3c), but the stone could not be removed easily intraoperatively. The cholecystectomy was performed. The gallbladder showed edematous changes due to inflammation, but was easily dissected. Thorough peritoneal lavage was performed, and the CBD perforation was resolved with a T-tube inserted through the perforation (Fig. 3d). Based on the preoperative examination and intraoperative findings, the patient was diagnosed as having a perforation of the CBD caused by a combination of congenital biliary dilatation with pancreaticobiliary maljunction, type II by Todani’s classification, and gallstone cholangitis/pancreatitis. Hematoxylin and eosin staining revealed that the resected specimen of the CBD wall was so destroyed that the muscular layer lacked continuity (Fig. 4a). Furthermore, immunohistochemical examination with anti-desmin antibody did not show the presence of smooth muscle in the tissue (Fig. 4b). Gallbladder wall revealed that dilated Rokitansky–Aschoff sinuses, mild muscular hyperplasia, and lymphocytic infiltration with hematoxylin–eosin staining.

**Fig.5:**
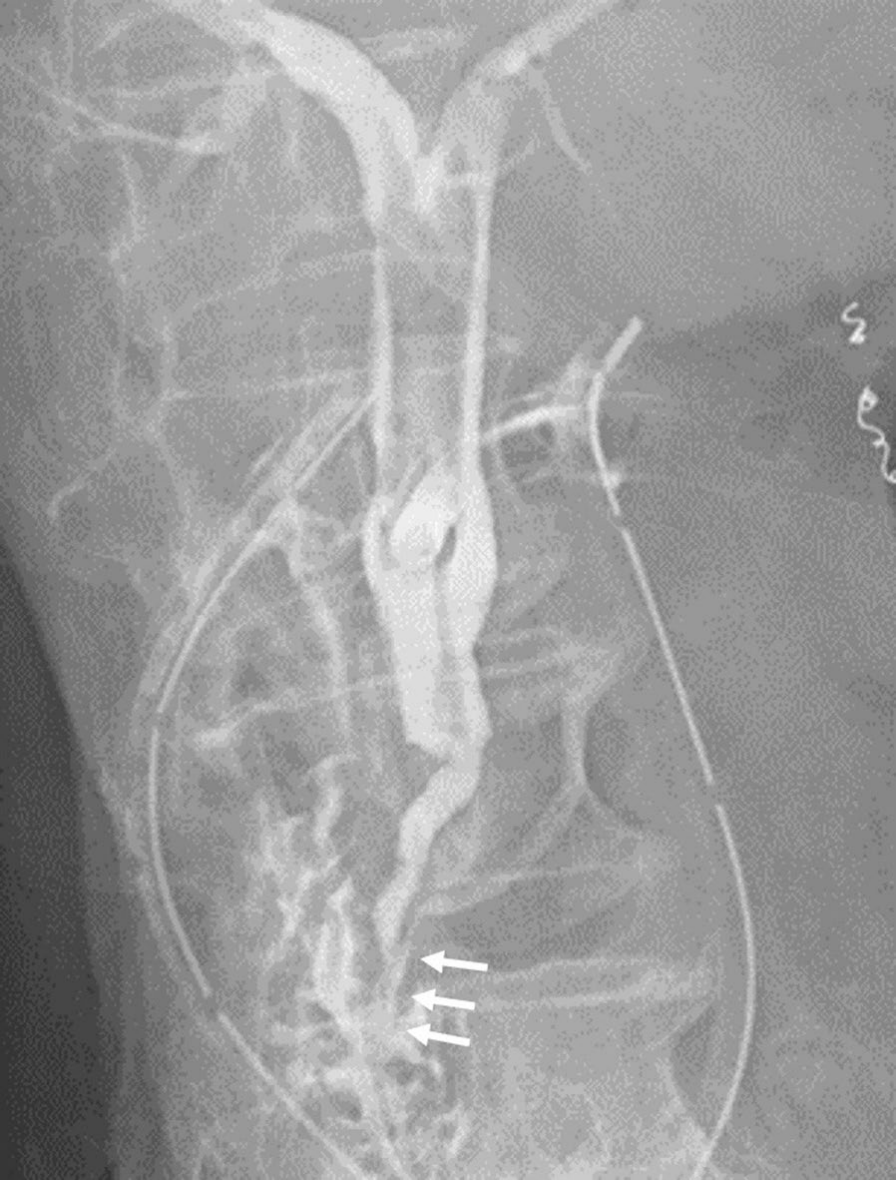
Postoperative T-tube cholangiogram. T-tube cholangiography on the 55th postoperative day confirmed that an impacted common bile duct stone had been removed naturally and that the long common channel of the pancreaticobiliary duct (arrow) clarified the diagnosis of pancreaticobiliary maljunction

The patient remained unstable and required inotropic agents, artificial respirator support and continuous hemodiafiltration in the intensive care unit until postoperative day 10. Furthermore, multiple additional drainage, and administration of antibacterial and antifungal agents were required for the refractory intra-abdominal and retroperitoneal abscesses. On day 16, active bleeding was observed at the abdominal wall around the T-tube, and hemostasis was achieved by transcatheter arterial embolization. On day 55, T-tube cholangiography revealed that the impacting CBD stone had disappeared naturally and, thus, the long common channel of the pancreaticobiliary duct and the diagnosed pancreaticobiliary maljunction could be observed (Fig. 5). After removal of the T-tube on day 136, she was transferred to the hospital for recuperation on day 152.

## Discussion

Spontaneous CBD perforation is one of the rare presentations of acute abdomen in infants and children and is extremely rare in adults. It was first described by Freeland in 1882 [13]. Either weakness in the wall of the bile duct or an increase in intraductal pressure or both have been suggested as the cause of the perforation [11]. Fragility of the bile duct wall is considered to be caused by choledochal cysts [14, 15], pancreatitis [15, 16], chronic infection [12], congenital weakness [11], ischemia [11], pancreatic reflux [11], and torsion of the gallbladder [11]. In contrast, reported causes of perforation with increased intraductal pressure include biliary sludge or stones [1–10], tumors [11], congenital stenosis of the ampulla of Vater [11], spasm of sphincter of Oddi [11], protein plugs [11], and parasites [11]. Diagnosis of the pathogenesis is difficult and delayed, because it sometimes occurs idiopathically.

In our search of PubMed between 2001 and 2021, we found 23 adult case reports of spontaneous extrahepatic CBD perforation with a detailed clinical course (Table 2) [1–12, 14–24]. We summarized these results to better understand the clinical features of the disease (Table 3). The summary showed that CBD perforation occurred more frequently in women. The mean age of the reported cases was 42 years (17–84 years). The mean duration of symptoms was approximately 2 weeks, which seems to be long compared to that of the usual acute abdomen, such as that caused by gastrointestinal perforations. We suppose that background diseases, which cause increased intraductal pressure or wall weakness, take relative longer to develop into a perforation. Choledocholithiasis was found in 10 cases (43.5%), most of which were accompanied with cholangitis or pancreatitis. These are considered to be typical cases in which the complex causes were consistent with increased intraductal pressure by stone impaction and wall weakening due to inflammation [1–10]. CBD perforation associated with pregnancy was observed in seven cases (30.4%), and all but one case developed in the third trimester. Although the relationship between pregnancy and CBD perforation is unclear, hemodynamic changes associated with higher pressure in the vena cava [2], raised intra-abdominal pressure [15], or global arteriolar spasm and impaired microcirculation due to preeclampsia [8] were mentioned as causes.

**Table 2:** Clinical features of 24 case reports (including our case) of spontaneous common bile duct perforation in adults searched from 2001 to 2021

Case	Author	Year	Age	Sex	Duration of symptoms (days)	Imaging modality for preoperative diagnosis	Paracentesis	Preoperative diagnosis
1	Balsarkar [2]	2001	21	F	1	US	Biliary fluid	N.D
2	Rege [17]	2002	55	M	2	US	Biliary fluid	N.D
3	Razman [18]	2004	36	M	14	N.D	Not performed	Perforated peptic ulcer
4	McGrath [3]	2005	34	F	1	N.D	Not performed	Appendicitis/perforation
5	Marwah [4]	2005	65	F	5	US	Not performed	Perforation of CBD
6	Talwar [19]	2006	21	F	3	US	Biliary fluid	Duodenal ulcer perforation
7	Joseph [14]	2008	28	F	5	US	Biliary fluid	N.D
8	Dabbas [5]	2008	20	F	56	US, CT	Not performed	N.D
9	Bhattacharjee [6]	2009	35	F	90	US, CT	Biliary fluid	Peptic or gallbladder perforation
10	Karvonen [20]	2009	67	M	7	CT	Biliary fluid	Not surgical case (ERBD placement)
11	Yaşar [21]	2009	38	F	14	US, CT	Not performed	N.D
12	Khanna [7]	2010	50	F	5	N.D	Biliary fluid	Peptic perforation
13	Laway [22]	2013	35	M	2	US, CT	Biliary fluid	Duodenal perforation
14	Paramhans [12]	2013	44	F	1	US	Not performed	N.D
15	Bowan [8]	2013	29	F	4	N.D	Biliary fluid	Peptic perforation
16	Singh [15]	2014	25	F	2	US	Not performed	N.D
17	Ishii [9]	2016	82	M	3	US, CT	Not performed	Hemorrhage/Colitis/Appendicitis
18	Pülat [16]	2016	36	F	7	US, CT	Not performed	Perforation of CBD
19	Subasinghe [10]	2016	66	F	3	US	Not performed	Peritonitis
20	Hamura [1]	2016	84	M	5	US, CT, (postoperative ERCP)	Biliary fluid	Bile peritonitis
21	Mohanty [11]	2017	17	F	3	US	Biliary fluid	Peptic perforation
22	Huda [23]	2017	40	M	90	US	Biliary fluid	Perforation of CBD
23	Amberger [24]	2018	28	F	N.D	US, CT, MRI, Scintigraphy	Biliary fluid	Perforation of CBD
24	Our case	2021	71	F	Unknown	US, CT	Biliary fluid	Perforation of CBD

Case	Causes	Surgical procedure (emergency)	Location of perforation	Outcome
1	Stone, pregnancy	T tube drainage	Below of the opening of the cystic duct	Alive
2	N.D	T tube drainage, cholecystectomy	Lateral wall of CBD inferior of the cystic duct	Alive
3	N.D	T tube drainage	Supraduodenal	Alive
4	Stone, pregnancy	Caesarean section	Junction of cystic duct (ERCP)	Alive
5	Stone	Cholecystectomy, choledochoduodenostomy	Supraduodenal	Alive
6	Pregnancy	T tube drainage	Supraduodenal	Alive
7	Pregnancy, choledochal cyst	T tube drainage, cholecystectomy	Supraduodenal	Alive
8	Stone, pregnancy	T tube drainage	Anterior surface	Alive
9	Stone	T tube drainage, cholecystectomy	Posterior wall	Alive
10	N.D	(ERCP case)	Common hepatic duct	Alive
11	N.D	T tube drainage, omentoplasty	Retropancreatic portion	Alive
12	Stone	T tube drainage	Supraduodenal	N.D
13	N.D	T tube drainage, cholecystectomy	Supraduodenal	Alive
14	Infection	T tube drainage, cholecystectomy	CBD at cystic junction	Alive
15	Stone, pregnancy	T tube drainage, cholecystectomy	2 parts: ① hepatic duct, ② supraduodenal	Alive
16	Choledochal cyst, pancreatitis, pregnancy	T tube drainage, cholecystectomy	2 parts: ① mid, ② supraduodenal	Alive
17	Stone	RTBD, cholecystectomy	Left hepatic duct	Alive
18	Pancreatitis	T tube drainage, cholecystectomy	Common hepatic duct	Alive
19	Stone	T tube drainage, cholecystectomy	CBD at cystic junction	Alive
20	Stone	Abdominal lavage only	N.D	Dead
21	N.D	T tube drainage, cholecystectomy	Supraduodenal	Alive
22	N.D	T tube drainage, cholecystectomy	Supraduodenal	Alive
23	N.D	(ERCP case)	Supraduodenal	Alive
24	Maljunction, stone, pancreatitis, cholangitis	T tube drainage, cholecystectomy	Supraduodenal	Alive

*US* ultrasonography, *CT* computed tomography, *ERCP* endoscopic retrograde cholangiopancreatography, *MRI* magnetic resonance imaging, *ERBD* endoscopic retrograde biliary drainage, *CBD* common bile duct, *N.D.* not described

**Table 3:** Summary of data from 23 case reports of spontaneous common bile duct perforation in adults searched from 2001 to 2021

Age (average)	41.6 ± 19.6 (17–84) years
Sex (M:F)	7:16
Duration of symptoms	14.7 ± 26.9 (1–90) days
Diagnosed preoperatively	17.4% (4/23cases)
With CBD stones	43.5% (10/23cases)
Pregnant	30.4% (7/23cases)
Location of perforation (SD: JCD: others)	50%:22.7%:27.3%
Postoperative bile leakage and abscess	30.4% (7/23cases)
Mortality	4.5% (1/22cases)

*CBD* common bile duct, *SD* Supraduodunal, *JCD* Junction of cystic duct

Although we could preoperatively diagnose CBD perforation with bilious ascites and discontinuity of the bile duct wall as proven by CT in our patient, only four cases (17.4%) in this review could be diagnosed preoperatively. In these cases, the authors reported that the loss of bile duct wall continuity on CT and high bilirubin levels in ascites were important diagnostic factors [4, 16, 23, 24]. Although most of the other cases were diagnosed by exploratory laparotomy, the perforation site in some cases could not be identified intraoperatively and required subsequent re-operation [5] or endoscopic retrograde cholangiopancreatography [3] for diagnosis. Notably, in a few cases, the perforation site could not be identified intraoperatively despite bile duct perforation being suspected preoperatively [1, 3]. In our summary, the most common site of perforation is the supra-duodenum (50%), followed by the junction of the cystic duct (22.7%). It may be helpful to observe these predominant sites when the perforation cannot be detected intraoperatively.

Surgical intervention is an effective treatment for CBD perforation as shown in our case. It is important to drain the abdominal contamination caused by the infected bilious peritoneal fluid. In most cases, T-tube drainage was followed by elective treatment for the causative diseases, such as endoscopic lithotomy for choledocholithiasis, resection of an extrahepatic bile duct, and hepaticojejunostomy for congenital biliary dilation. In cases diagnosed as idiopathic after detailed evaluation, the T-tube was removed without additional treatment. As a result of this review, we recommend prompt and appropriate peritoneal and biliary drainage in the unstable phase with peritonitis, followed by accurate assessment of the necessity of additional treatment for the background disease in the stable phase.

Postoperatively, our patient suffered from refractory intra-abdominal and retroperitoneal abscesses and the hemorrhagic shock due to the rupture of an aneurysm formed along the T-tube fistula. Our review indicated that postoperative complications such as bile leakage or intraabdominal abscess were reported in 30.4% of the cases. Compared to the patients in the literature review, our patient required more time for treatment of postoperative complications, and we considered that one of the reasons was due to the mechanism of pancreaticobiliary maljunction. Pancreaticobiliary maljunction is generally accepted as a congenital condition in which the pancreatic and bile ducts join anatomically outside the duodenal wall. Because the action of the sphincter of Oddi does not affect the pancreaticobiliary junction, pancreaticobiliary reflux occurs. As a result, various pathologic conditions, such as obstruction of bile and pancreatic outflow, carcinoma, or inflammation, can occur [25]. In the present case, abscess formation and bleeding from the abdominal wall were considered specific postoperative complications, because pancreatic enzymes were activated by mixing with bile due to reflux of pancreatic juice into the bile duct. The main cause of aneurysms associated with pancreatic fistulas is corrosion and weakening of the vessel wall caused by leaking activated pancreatic juice [26]. The abdominal wall aneurysm in our case might have occurred due to a mechanism similar to that described above.

One of the clinical questions in the presented case is whether the diverticulum-like imaging finding of CBD was due to the coexistence of congenital biliary dilation, type II by Todani’s classification, or secondary changes associated with perforation. Glenn et al. suggested that congenital biliary dilatation that forms a diverticulum may be due to hypoplasia of the bile duct wall muscularis [27]. Previous reports have shown the presence of a thinning muscularis [28]. We thought that the pathological evaluation of the resected specimen might help to distinguish this. Hematoxylin and eosin staining and immunohistochemical examination with anti-desmin antibody were performed, but neither showed a muscular layer. Even if the muscular layer were present, it would have been difficult to distinguish it because of the strong tissue destruction caused by inflammation. As a result, although we finally judged that congenital biliary dilation could not be definitely diagnosed, it could be a possibility based on the imaging findings.

## Conclusion

This is the first case report of spontaneous CBD perforation accompanied with pancreaticobiliary maljunction. Early surgical treatment and appropriate perioperative management prevented mortality in our patient. Spontaneous CBD perforation should be considered in the differential diagnosis if the perforation cannot be identified during exploratory laparotomy.

## Data Availability

All data generated or analyzed during this study are included in this published article.

## Abbreviations

CBD: Common bile duct
CT: Computed tomography
